# Groups of Geomicrobiological Indicators Are Spread across Gas-Hydrate and Non-Gas-Hydrate Areas in the Northern Part of the Sea of Japan

**DOI:** 10.3390/biology11121802

**Published:** 2022-12-12

**Authors:** Anna L. Ponomareva, Alena I. Eskova, Renat B. Shakirov, Nadezhda S. Syrbu, Aleksey A. Legkodimov, Roman A. Grigorov

**Affiliations:** Il’ichev Pacific Oceanological Institute, Far Eastern Branch of the Russian Academy of Sciences, 690022 Vladivostok, Russia

**Keywords:** microorganisms, Sea of Japan, bottom sediments, oil-oxidizing strains, aerobic and anaerobic destruction of hydrocarbons, bioindicator genes, gas hydrates

## Abstract

**Simple Summary:**

The bioindication of oil and gas fields is an important task in geomicrobiology. Our study expands the field of geomicrobiological methods and allows us not only to suggest the possibility of the presence of an oil and gas deposit but also its type. We used cultural and molecular genetic methods to study microorganisms, in particular their ability to oxidize hydrocarbons under aerobic and anaerobic conditions. We carried out a comparative analysis of cultured and molecular methods for the bioindication of oil and gas deposits to detect deposits and describe the relevant types(traditional and gas-hydrate). The most promising method in this area was the one used for assessing the anaerobic degradation of hydrocarbons. Studying the *mcrA*, *pmoA*, *dsrB*, *alkBB*, *bssA* and *masD* biomarker genes made it possible to identify areas with different types of deposits based on the distribution of the *alkBB*, *bssA* and *masD* genes. The *bssA* and *masD* genes predominated in the gas-hydrate region. With the help of cultured methods, it was possible to describe the type of deposits based on taxonomic diversity (*Rhodococcus* spp.). In addition, the ability of aerobic and facultative anaerobic hydrocarbon-oxidizing microorganisms to anaerobically utilize hydrocarbons was described using cultured methods.

**Abstract:**

The bioindication of oil and gas fields is a field of geomicrobiology that is mainly devoted to the detection of hydrocarbon-oxidizing microbial indicator species or functional genes in total DNA. However, it appears promising to use the physiological properties of microorganisms detection deposit type of hydrocarbons, in particular their ability to oxidize hydrocarbons under aerobic and anaerobic conditions. In this study, the most promising approach in this area was the method used for assessing the anaerobic degradation of hydrocarbons. When comparing molecular genetics and cultured methods of bioindication, it can be concluded that molecular biomarkers of functional genes for the anaerobic destruction of hydrocarbons (*masD*) make it possible to separate areas with traditional and gas-hydrate types of deposits. Using cultured methods, we found that representatives of the Nocardiaceae family of the phylum *Actinomycetota* were tied to the areas where gas hydrates were found. The ability of aerobic and facultative anaerobic hydrocarbon-oxidizing microorganisms to anaerobically utilize hydrocarbons was determined with cultured methods. For the first time, this ability was revealed for the genera *Stenotrophomonas*, *Psychrobacter*, *Micrococcus* and *Peribacillus*. The wide distribution of this ability that we found in strains isolated from both study regions suggests its prominent role in the destruction of hydrocarbons in marine sediments.

## 1. Introduction

Oil and gas deposits in sea bottom sediments have a strong impact on the formation of microbiomes, especially on bacteria capable of oxidizing methane and other hydrocarbons [1,2,3,4,5]. Communities of microorganisms found in such areas have specific functions and are rigidly structured both in depth and in distance from the seep [3,6,7,8,9]. The high ecological plasticity and biodiversity of hydrocarbon-oxidizing bacteria make them able to switch to the consumption of methane and other hydrocarbons, depending on the environmental conditions [8,9,10,11,12,13,14,15]. However, oil and gas deposits are different types due to their geological characteristics, and, depending on this, have different effects on the formation of the microbial community. Gas-hydrates are relatively new types of hydrocarbon deposits that presumably contain large volumes of gas [6,16,17]. They are formed under the conditions of a combination of low temperature and high pressure in marine bottom sediments with high concentrations of natural gas in the sedimentary environment and on the bottom surface. There have been few reports of the phyla of non-culturable microorganisms found only in areas where gas hydrates have been found [18,19]. The use of bioindication methods allows for comprehensively assessing the total impact of simultaneous processes taking place in the areas of gas flows at the bottom of an ocean.

The main methods in the field of bioindication at present are the detection of functional genes involved in the biodegradation of hydrocarbons and the description of properties of bioindicator species [13,19,20,21,22,23,24]. Due to the plasticity of bioindicator methods, we assume that they can be used not only for the detection of oil and gas deposits but also for more detailed descriptions of such deposits, for example, for the identification of traditional and gas-hydrate deposits, without direct access to the surface of gas hydrates [18,19].

Comparison of the range of applications of different approaches to bioindication will allow us to identify the most promising bioindication methods for the assessment of the distribution of functional genes and taxonomic or physiological characteristics of bioindicator microorganisms [25,26].

The processes of bacterial oxidation of hydrocarbons directly in oil and gas reservoirs are significantly different from the processes occurring outside the deposit. The main feature of the environmental conditions in the area of oil and gas occurrence is the anaerobic environment. We made an assumption that the ability of bacteria to oxidize oil under anaerobic conditions may be a significant bioindicator feature [27,28].

Thus, the purpose of this study was to determine the possibility of identifying not only an oil and gas deposit but also its type (traditional or gas hydrate) by comparing various bioindicator approaches (molecular–genetic, cultural). As the first step, the distribution of the most commonly used genes responsible for the biodegradation of hydrocarbons (*alkBB*, *masD* and *bssA*) and markers for the synthesis and oxidation of methane (*pmoA* and *mcrA*) that are associated with them in the carbon cycle were assessed [21,22,29,30,31]. In the second stage, an analysis of the distribution of bioindicator species of microorganisms depending on the type of deposit was carried out. In the third stage, the ability for anaerobic oxidation was studied in oil-oxidizing strains isolated from areas with different types of oil and gas deposits [32,33]. This study enabled us to estimate the methods and possible volumes of the anaerobic oxidation of hydrocarbons, which occurs in bottom sediments in areas of gas fields. Comparative analysis of all stages allowed us to describe the possible methods of bioindication of different types of deposits.

## 2. Materials and Methods

### 2.1. Study Area

**Figure 1:**
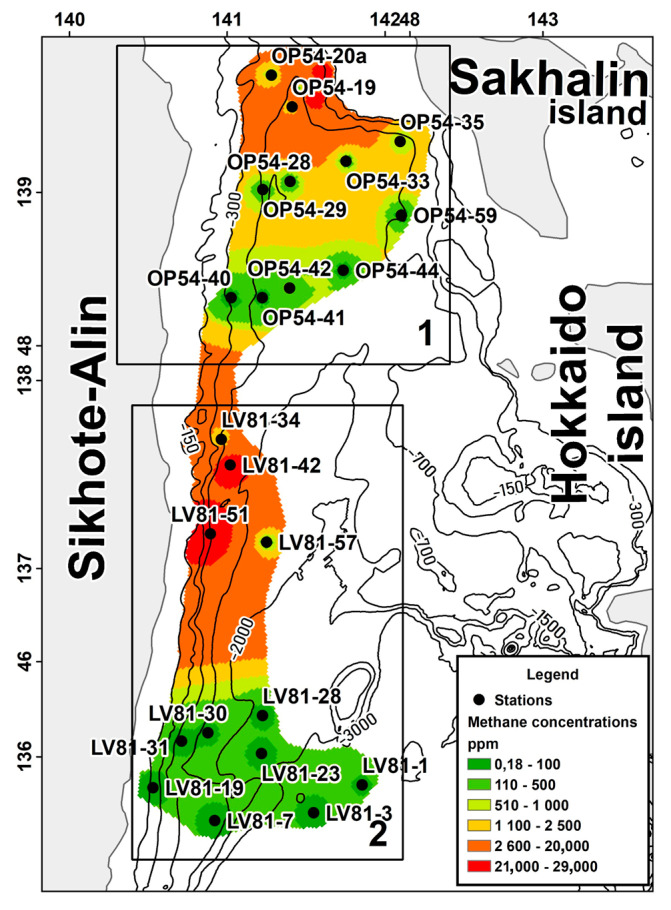
Study area. Methane content (ppm) in bottom sediments of the studied areas of the northern part of the Sea of Japan. (1) Gas-hydrate area; (2) non-gas-hydrate area.

The study area is located in the northern part of the Sea of Japan and includes two zones. The first one is characterized by the presence of gas hydrates within the south-western slope of Sakhalin Island. Gas hydrates were found near stations OP54-20a and OP54-19 near Sakhalin Isl. The second zone is located closer to the Central Basin of the Sea of Japan (the region of the northern closure of the Central Basin) (Figure 1).

The average concentration of methane in the non-gas-hydrate area is significantly higher than the same indicator in the gas-hydrate area. In addition, the non-gas-hydrate region is deeper than the gas-hydrate region.

Stations where gas hydrates were directly detected were not included in the study. An analysis of the methane content was performed on a two-channel gas chromatograph “Crystallux-4000M” (CJSC “Meta-Khrom”, Yoshkar-Ola, Russia) equipped with a flame ionization detector, with ionization flux and thermal conductivity sensors and a sensitivity level of 10^−6^%.

### 2.2. Sample Selection

Core samples were obtained by shallow drilling using a stainless steel gravity sampler with an internal diameter of 130 mm and a length of 600 cm (Table 1).

We used samples from the upper part of the restored layer of sea bottom sediments in the northern part of the Sea of Japan, taken during cruises OP54 of the R/V “Akademik A.I. Oparin” (2017) and LV81 of the R/V “Akademik M. A. Lavrentiev” (2018) to study the distribution of microorganisms in bottom sediments associated with gas discharge.

### 2.3. Stage 1 of the Study. Detection of Functional Genes in Marine Sediments

#### 2.3.1. DNA Isolation

A modified method proposed by Marmur [34] was applied to isolate total DNA from bottom sediments and obtained strains. A total of 1 g of sea bottom sediment was ground in a sterile mortar for 5 min to free the cells from the core. Next, the sediment was placed in 1 mL of TE buffer (10 mM Tris pH 8.0, 1 mM EDTA). A 20% SDS solution was added to the suspension to a final concentration of 1–2% and thoroughly mixed for 5 min in a vortex. This suspension was incubated for 30 min at a temperature of 37 °C in a thermostat. After incubation, equal volumes of a mixture of phenol and chloroform were added to each tube, thoroughly mixed in a vortex, and centrifuged for 10 min at 15,000 rpm. The upper phase was transferred to a new test tube, and an equal volume of a mixture of phenol and chloroform at a ratio of 1:1 was again added, mixed, and centrifuged for 10 min at 15,000 rpm. The upper phase was transferred to a new tube, extracted with an equal volume of chloroform, and centrifuged for 10 min at 15,000rpm. Next, 5M NaCl and 3 volumes of chilled 96% ethanol were added, thoroughly mixed, and placed in a freezer at −40 °C for at least 2 h. The sample was then centrifuged again for 15 min at 15,000 rpm. The excess alcohol was removed, and the DNA precipitate was air-dried.

#### 2.3.2. PCR

The determination of the presence of functional genes is presented in Table 2 (Amplifier Dtprime-5, DNA Technology Moscow, Russia). The results were analyzed using the software supplied with the instrument RealTime_PCR v7.9 (Moscow, Russia).

### 2.4. Stage 2 of the Study: Diversity of Cultivated Bioindicator Hydrocarbon-Oxidizing Microorganisms

Our aim was to detect biodegradative functional genes:

1. 1-monooxygenase (*alkBB*) gene, which is a key catabolic gene of alkane degradation [28];
2. The mechanism of anaerobic alkane activation is the addition of alkanes to fumarate by (1-methylalkyl) succinate synthase (*mas*). Here, we studied the *masD* gene in the catalytic subunit of the enzyme [20,35];
3. The *bssA* gene, coding for benzylsuccinate synthase, the key enzyme of anaerobic toluene degradation [30];
4. Methanotrophs oxidize methane using methane monooxygenase (MMO) enzymes, and the subunit gene of the most common MMO *pmoA* was selected [22];
5. Methanogenic microorganism gene, coding metagenomic methyl-coenzyme M reductase alpha subunit (*mcrA*) [31].

#### 2.4.1. Culture Methods

To create accumulative cultures of oil-oxidizing microorganisms, 3 media were used (Table S1).

As the only carbon source, ESPO crude oil was used at a ratio of 4%.

A total of 1 g of bottom sediments was added to each medium and cultivated at 25 °C for 30 days. Pure cultures were isolated using standard microbiological methods (depleting stroke method and Drygalski method).

At least 1 g of bottom sediments was added to the selective medium and cultured at a temperature of 20 °C for 3 weeks. Pure cultures were isolated using the streak plate method.

#### 2.4.2. Identification of the Researched Isolates

The isolation of total DNA from samples of the bottom sediments and chromosomal DNA of pure cultures was carried out using the modified Marmur method [34]. The identification was carried out by sequencing a highly conservative 16S rDNA site with the Sanger method using the BigDye v3.1 kit on the ABI 3130xl genetic analyzer (ThermoFisher Scientific, Waltham, MA, USA); sequencing was carried out by the company Syntol LLC, Moscow, Russia. Universal bacterial primers 11F (5′ AGTTTGATCATGGCTCAG 3′) and 1100R (5′ GGGTTGCGCTCGTTG3′) were used for sequencing [36]. The obtained sequences were identified up to individual operational taxonomic units (OTUs) at a similarity level of 98%.

Determination of the level of hydrocarbon biodegradation was conducted using the fluorometric method.

#### 2.4.3. Phylogenetic Analysis

Phylogenetic analysis was performed by searching for homologous sequences in the International Data Bank (GenBank) using the BLAST program [37] (http://www.ncbi.nlm.nih.gov/blast (accessed on 6 July 2022)). The resulting sequences were checked for the presence of chimeras using the Pintail 1.1 program (http://www.bioinformatics-toolkit.org/Web-Pintail/ (accessed on 8 June 2022)). Sequence editing was performed using the BioEdit editor; sequence alignment was performed using the algorithm of the CLUSTAL W program (http://www.genebee.msu.su/clustal (accessed on 6 July 2022)). Phylogenetic trees were built using the Robust Phylogenetic analysis software package presented on the website http://www.phylogeny.fr/ (accessed on 10 July 2022) [38].

Stage 3 of the study. The ability of cultivated microorganisms to utilize hydrocarbons under aerobic and anaerobic conditions.

The microorganisms isolated at the previous stage of the study were cultivated on modified Voroshilova–Dianova medium under aerobic and anaerobic conditions at a temperature of 25 °C for a week. Anaerobic conditions were created by replacing the atmosphere with nitrogen.

#### 2.4.4. Analysis of Hydrocarbon Biodegradation

Analysis of the degree of hydrocarbon biodegradation was carried out using fluorometric analysis. This method is based on the extraction of hydrocarbons with hexane from the sample and the measurement of the fluorescence intensity of the extract on the Fluorat-02-Panorama liquid analyzer according to a standardized method, which is based on the extraction of petroleum products with hexane and purification of the extract if necessary, which is followed by measurement of the fluorescence intensity of the extract. The cultivation of the studied strains was carried out in liquid Voroshilova–Dianova medium, containing oil hydrocarbons making up no more than 1% of the total volume of the medium. Nitrogen-purged Hungate tubes were used to create anaerobic conditions.

The media inoculated with microorganisms were cultivated under aerobic and anaerobic conditions for 14 days at a temperature of 25 °C. Then, the oil products were extracted with hexane. The measurement was carried out in the short-wavelength region (270–290 nm), and fluorescence was recorded in the region 300–330 nm, which made it possible to reduce the dependence of the analytical signal on the composition of the hydrocarbon mixture.

#### 2.4.5. Statistical Analysis

The nucleotide sequences of the 16S rRNA gene fragments of the isolated strains of oil-oxidizing bacteria were registered in the GenBank database. The diversity of the isolated strains was determined using the Shannon and Simpson indices [11].

Microsoft Office Excel 2007 spreadsheets were used for calculations and systematization of the data obtained during the cruise. The data obtained immediately after processing were entered into the ArcGIS 10.4 geoinformation application, using the Geostatistical Analyst module, for interpretation. Statistical analyses and data visualizations were created using R Version 3.3.1 (https://cran.r-project.org/bin/windows/base/old/3.1.1/ (accessed on 12 September 2022)).

## 3. Results

### 3.1. Distribution of Functional Bioindicator Genes in Bottom Sediments of Gas-Hydrate Area and Non-Gas-Hydrate Area of the Sea of Japan

#### 3.1.1. Gas-Hydrate Area

**Figure 2:**
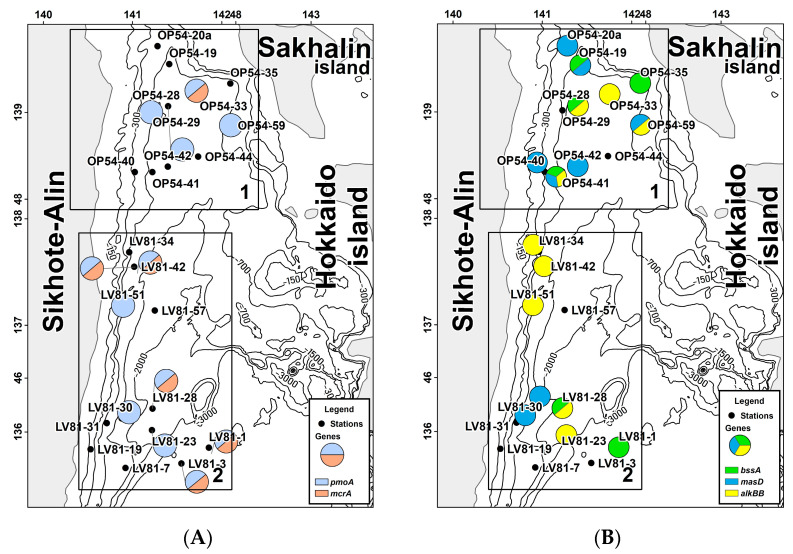
Distribution of functional bioindicator genes in bottom sediments of the northern part of the Sea of Japan. The stations are divided into (1) the gas-hydrate area and (2) the non-gas-hydrate area. (**A**) Methane cycle; (**B**) Biodegradation of hydrocarbons.

At four stations out of 12, bioindicator genes of methanotrophic and methanogenic prokaryotes were found. Stations were located in the central part of the area at depths from 600 to 1100 m. *mcrA* genes were recorded at one of the deepest stations in the gas-hydrate region (1094 m). Bioindicator genes of the methane cycle were found at stations with methane concentrations closest to the average, except for the maximum value in the gas-hydrate region (about 100 ppm). At station OP54-59, *pmoA* was detected at a station with a minimum methane concentration of 0.002 ppm. At the stations located near the gas-hydrate discovery region, *mcrA* and *pmoA* were not recorded (Figure 2A,B).

In the gas-hydrate region, at eight stations out of 12, functional genes responsible for the degradation of hydrocarbons were recorded. The *masD* gene was encountered with the highest frequency (five stations out of eight). At three stations, two bioindicator genes were detected, and at one station, all three functional genes under study were detected. One gene was found at four stations (*masD* at three stations, *bssA* at one station) (Figure 2B).

According to the detection of bioindicator genes, the gas-hydrate region can be divided into three parts (Figure 2B).

Stations OP54-19, 20a, and 35 are located near Sakhalin Island near the location where gas hydrates were found. They are equally represented by the *masD* and *bssA* genes. At the same time, *bssA* was registered at the station with the highest content of methane in the gas-hydrate region.

Stations OP54-29, 33, and 59 are located in the center of the gas-hydrate region. *alkBB* genes were recorded only at these stations.

Stations OP54-40, 41, and 42 are located at depths from 700 to 1100 m. The *masD* gene was detected at all stations. At these stations, the methane content was observed to be below the average for the gas-hydrate region.

#### 3.1.2. Non-Gas-Hydrate Area

In the study of bioindicator genes involved in the methane cycle in the non-gas-hydrate region, *pmoA* was found at eight stations out of 12. These stations were located at different depths and had a wide range of methane concentrations, including the maximum. The *mcrA* gene was recorded at four stations. Two of them were located at depths of 300 to 700 m and had the highest methane concentrations. The other two stations at which *mcrA* were found were in the deepest part of the test site.

In the non-gas-hydrate area, at eight out of 11 stations, bioindicator functional genes involved in the oxidation of hydrocarbons were recorded. In this area, *alkBB* was encountered with the highest frequency at five stations out of 11. The *masD* gene was encountered at two stations, as was *bssA*. At stations with the highest methane contents in the area, *alkBB* was detected, and other studied functional genes *masD* and *bssA* were detected at stations with methane contents slightly exceeding the background values.

The features of the non-gas-hydrate region were the predominance of *alkBB* and the detection of only one bioindicator gene responsible for the utilization of hydrocarbons at the station. In the non-gas-hydrate region, only at one station, LV81-28, the *alkBB* and *bssA* genes were detected.

#### 3.1.3. Comparison of Gas-Hydrate and Non-Gas-Hydrate Regions

In the gas-hydrate region, the bioindicator genes of the methane cycle were recorded less frequently than in the non-gas-hydrate region. In both studied zones, the bioindicator gene of methanogenic prokaryotes was found in areas with the greatest depth, regardless of the methane content in the bottom sediments. In the gas-hydrate region, the bioindicator gene of methanotrophic bacteria was found at stations with the average and lowest methane contents in the region as well as in the non-gas-hydrate region, regardless of the methane content at the station.

The genes for the anaerobic oxidation of linear hydrocarbons predominated in the gas-hydrate region, and vice versa, in the non-gas-hydrate region, genes for aerobic oxidation of hydrocarbons predominated. The detection of *pmoA* in both areas occurred at the same stations and near the stations at which *alkBB* was recorded. Thus, the detection of genes responsible for the aerobic (non-gas-hydrate region) and anaerobic (gas-hydrate region) degradation of hydrocarbons (*alkBB* and *masD*) had the greatest bioindicator value for different regions.

### 3.2. Taxonomic Diversity of Hydrocarbon-Oxidizing Bacteria Isolated from the Upper Part of the Oxidized Layer of Bottom Sediments in the Areas of Presence and Absence of Gas Hydrates in the Northern Part of the Sea of Japan

#### 3.2.1. Gas-Hydrate Area

In the gas-hydrate region, 37 strains were isolated from the surface layer of bottom sediments at eight stations out of 12. The predominant species in this area are representatives of the genera *Pseudomonas*, *Stenotrophomonas* and *Psychrobacter* (11, 7, and 7 strains, respectively). Eight strains were unique to their area. Of these, four belonged to the widest known genus of hydrocarbon-degrading bacteria, *Rhodococcus*. The remaining four strains were assigned to *Nesterenkonia lutea, Peribacillus simplex, Promicromonospora xylanilytica* and *Nocardioides dokdonensis*. *Rhodococcus* ssp. was isolated near the location where gas hydrates were found, as were other unique strains (Figure S1).

#### 3.2.2. Non-Gas-Hydrate Area

From the surface layer of the bottom sediments of eight stations out of 12 in the non-gas-hydrate area, 17 strains of hydrocarbon-oxidizing bacteria were isolated. At station OP54-57, five strains were isolated; at other stations, from one to three strains were isolated, the largest number of which belonged to the genus *Psychrobacter* (five strains). The representatives of *Stenotrophomonas* and *Pseudomonas* belonged to four and three strains, respectively. The following strains were unique for this region: *Robertmurraya kyonggiensis*, *Curtobacterium oceanosedimentum*, *Brevibacillus nitrificans* and *Micrococcus* sp. (Figure S2).

#### 3.2.3. Comparison of Gas-Hydrate and Non-Gas-Hydrate Regions

The largest number of common species for the two study areas belongs to the phylum Gammaproteobacteria. The gas-hydrate region is characterized by a significantly large number of isolates. Thus, 37 of 54 strains were isolated in the gas-hydrate region (nine genera), and 17 strains (eight genera) were isolated in the non-gas-hydrate region. Representatives of the genera *Bacillus*, *Pseudomonas*, *Stenotrophomonas*, and *Psychrobacter* were found in both study areas. In the gas-hydrate region, the following were unique: *Rhodococcus*, *Nesterenkonia*, *Promicromonospora*, *Nocardioides* and *Peribacillus*. In the non-gas-hydrate region *Brevibacillus*, *Micrococcus*, *Curtobacterium* and *Robertmurraya* were unique.

**Figure 3:**
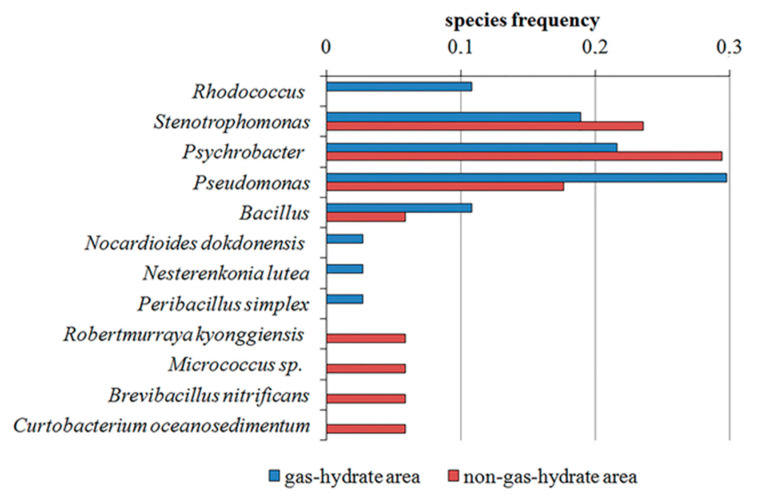
The frequency of occurrence of genera and species in the gas-hydrate and non-gas-hydrate areas.

Representatives of the genus *Pseudomonas* occurred with the greatest frequency in the gas-hydrate region. In the non-gas-hydrate region, there was no clear predominance of one genus; *Pseudomonas*, *Stenotrophomonas* and *Psychrobacter* were found in the greatest numbers. The gas-hydrate region is characterized by a significantly large number of isolates (Figure 3).

**Figure 4:**
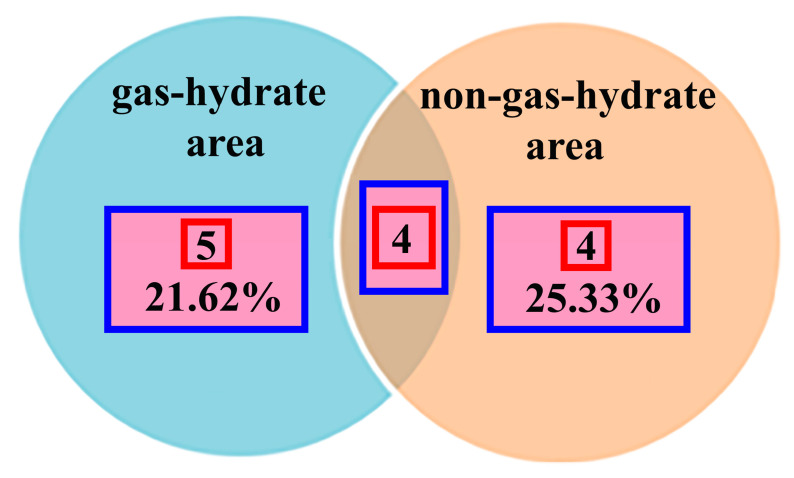
Comparison of the distribution of genera in the gas-hydrate and non-gas-hydrate regions. Red square—number of genera, blue square—number of unique genera for each zone as a percentage of the total number.

All strains belonging to the phylum Actinomycetota were unique for the gas-hydrate region. At the same time, representatives of the Nocardiaceae family were found only in the gas-hydrate region. The family is represented by five strains: *Nocardioides* (1), *Rhodococcus* (4), which are assigned to two genera. At the same time, the frequency of the occurrence of species unique to this region in the gas-hydrate and non-gas-hydrate regions is comparable (21.62 and 25.33%, accordingly) (Figure 4).

The Shannon and Simpson indices were calculated (Table 3) to assess biodiversity and dominance. The analysis of the indices obtained shows that the Shannon genera diversity index in the gas-hydrate region was slightly lower than that in the non-gas-hydrate region. The Simpson index in the gas-hydrate region was slightly higher than in the non-gas-hydrate region. At the level of genera, the studied areas were characterized by insignificant microbial diversity.

The Jaccard coefficient was calculated to assess the similarity and difference in the taxonomic composition of the isolated strains between the studied areas. When comparing the number of species in gas-hydrate and non-gas-hydrate regions, the Jaccard similarity coefficient is quite low (0.17), which indicates a large difference in the taxonomic composition at the level of cultivated species.

### 3.3. Description of the Ability of the Studied Strains to Biodegrade Oil Hydrocarbons under Aerobic and Anaerobic Conditions

#### 3.3.1. Gas-Hydrate Area

The largest number of strains isolated in the gas-hydrate region had the ability to utilize hydrocarbons under both aerobic and anaerobic conditions. Only 14 out of 37 strains did not have this property. These strains belonged to the genera *Rhodococcus*, *Stenotrophomonas*, *Psychrobacter*, *Pseudomonas*, *Nocardioides dokdonensis* POI OP54-35/al26-1, and *Nesterenkonia lutea* POI OP54-19/165. The largest number of strains incapable of utilizing hydrocarbons under anaerobic conditions belonged to the genus *Psychrobacter* (three out of seven strains). All representatives of the genus *Bacillus* possessed the ability to utilize oil hydrocarbons both in the presence and absence of oxygen.

The degree of the biodegradation of hydrocarbons by strains capable of this activity only under aerobic conditions varied in the range 51.98 to 97.38% of the initial concentration of hydrocarbons. For strains capable of oxidizing hydrocarbons both in the presence and absence of oxygen under aerobic conditions, the same indicator ranged from 56.07 to 88.22% of the initial concentration of hydrocarbons. The degree of the degradation of hydrocarbons under anaerobic conditions was significantly higher than the same indicators in aerobic conditions (from 80.95 to 92.22% of the initial concentration of hydrocarbons) (Table S2).

Among the strains capable of only aerobic degradation of hydrocarbons, representatives of the genus *Psychrobacter* were the most active, while *Stenotrophomonas* strains were the least active.

In addition, only in *Psychrobacter* strains, similar indicators of the intensity of hydrocarbon degradation under aerobic conditions were observed in strains capable of only aerobic oil degradation and strains capable of both aerobic and anaerobic oil degradation.

In all other strains tested, carbohydrate consumption under anaerobic conditions was significantly higher than under aerobic conditions. The difference between the aerobic and anaerobic utilization of oil was more than 10%. In representatives of the genus *Stenotrophomonas*, this difference was the smallest.

**Figure 5:**
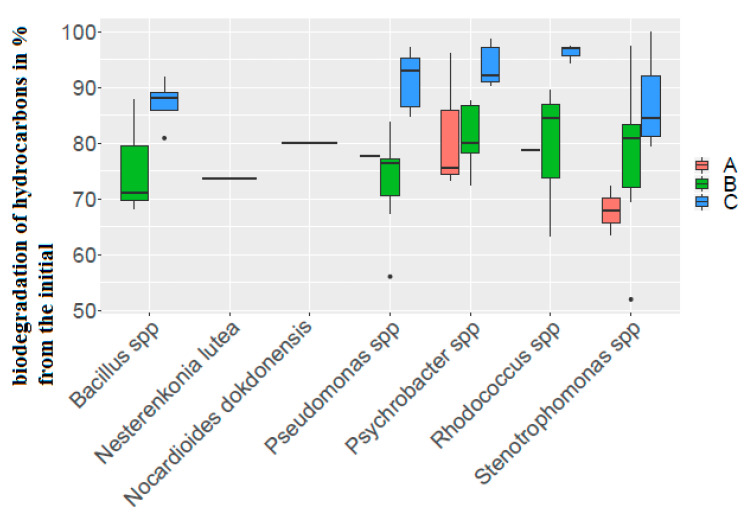
Biodegradation of hydrocarbons by strains isolated from the gas-hydrate region. (A) The degree of biodegradation of oil by strains capable of utilizing oil only under aerobic conditions; (B) the degree of oil biodegradation by strains capable of utilizing oil both under aerobic and anaerobic conditions (aerobic conditions); (C) the degree of oil biodegradation by strains capable of utilizing oil both under aerobic and anaerobic conditions (anaerobic conditions). The corresponding data can be found in Table S2.

Representatives of the genus *Rhodococcus* have a wide scatter of data on the degradation of hydrocarbons under aerobic conditions and very little under anaerobic conditions (Figure 5).

#### 3.3.2. Non-Gas-Hydrate Area

Only three out of 17 strains, *Psychrobacter pacificensis* POI LV81-57/al 379 (2), *Stenotrophomonas rhizophila* strain POI LV81-28/173-3(3), and *Curtobacterium oceanosedimentum* strain POI LV81-23/149-3(2), isolated in the non-gas-hydrate area, did not have the ability for anaerobic oil degradation. The degree of oil biodegradation by these strains ranged from 75.56 to 86.33% of the initial amount of hydrocarbons.

Strains capable of utilizing oil under aerobic and anaerobic conditions in the presence of oxygen demonstrated a lower ability to degrade oil than strains capable of utilizing oil only under aerobic conditions (from 53.17 to 69.33% of the initial amount of hydrocarbons). At the same time, under oxygen-free conditions, the same indicator for the tested strains was quite high from 85.06 to 100% (out of the limits of determination) of the initial amount of hydrocarbons. The maximum degree of biodegradation was observed in the strain *Stenotrophomonas maltophilia* POI LV81-57/379-1 (Table S3).

Unfortunately, most of the genera had only one representative in the sample of strains isolated from the bottom sediments of the non-gas-hydrate region, which made it difficult to assess the properties of the genus.

As well as in the gas-hydrate region, the difference between anaerobic and anaerobic oil consumption by strains was about 10%. In contrast to the gas-hydrate region, this indicator in representatives of the genus *Stenotrophomonas* was significantly higher and corresponded to other studied strains.

**Figure 6:**
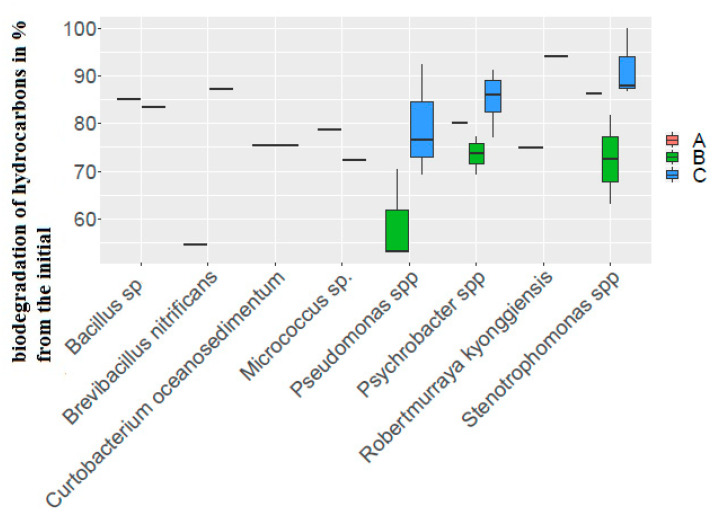
Biodegradation of hydrocarbons by strains isolated from the non-gas-hydrate region. (A) The degree of biodegradation of oil by strains capable of utilizing oil only under aerobic conditions; (B) the degree of oil biodegradation by strains capable of utilizing oil both under aerobic and anaerobic conditions (aerobic conditions); (C) the degree of oil biodegradation by strains capable of utilizing oil both under aerobic and anaerobic conditions (anaerobic conditions). The corresponding data can be found in Table S3.

Representatives of the genus *Pseudomonas* isolated in the non-gas-hydrate region utilized hydrocarbons more slowly under both aerobic and anaerobic conditions. The decrease in indicators was about 5 to 8%. For representatives of the *Psychrobacter* and *Stenotrophomonas* genera, the values of the degree of hydrocarbon degradation for strains isolated from the gas-hydrate and non-gas-hydrate regions were close (Figure 6).

#### 3.3.3. Comparison of Gas-Hydrate and Non-Gas-Hydrate Regions

In the non-gas-hydrate region, strains capable of utilizing oil only with the presence of oxygen accounted for 17.6% of the total number of strains isolated in the region. In the gas-hydrate area, the same figure was significantly higher at 37.8%.

In both study areas, some representatives of the genera *Stenotrophomonas* and *Psychrobacter* did not have the ability to utilize oil hydrocarbons under anaerobic conditions. Only in the gas-hydrate region, strains belonging to the genera *Pseudomonas*, *Rhodococcus*, *Nesterenkonia* and *Pseudomonas* did not have the same property. In the non-gas-hydrate area, *Curtobacterium*, *Rhodococcus* and *Nesterenkonia* were unique in their area of discovery.

**Figure 7:**
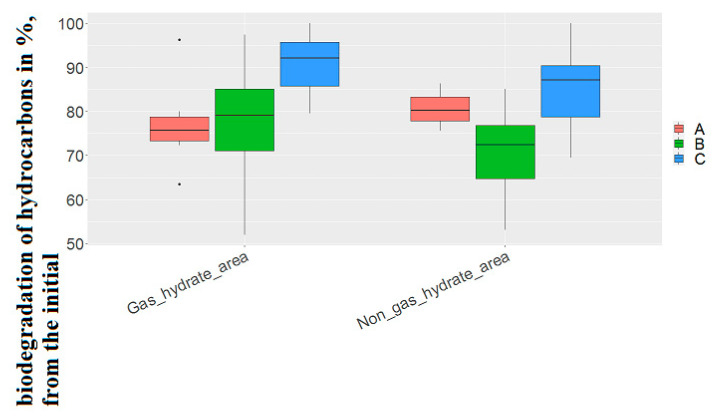
Biodegradation of hydrocarbons by strains isolated from gas-hydrate region and non-gas-hydrate region. (A) The degree of biodegradation of oil by strains capable of utilizing oil only under aerobic conditions; (B) the degree of oil biodegradation by strains capable of utilizing oil both under aerobic and anaerobic conditions (aerobic conditions); (C) the degree of oil biodegradation by strains capable of utilizing oil both under aerobic and anaerobic conditions (anaerobic conditions).

On average, strains capable of utilizing hydrocarbons under both aerobic and anaerobic conditions in the gas-hydrate region had higher values of the degree of hydrocarbon biodegradation (77.22 ± 8.93 and 99.87 ± 6.05 in the gas-hydrate region, 70.1 ± 10.42 and 85.1 ± 8.6% of the initial concentration of hydrocarbons, respectively), and strains capable of oxidizing oil only under aerobic conditions, conversely, had lower values in the non-gas-hydrate region (76.8 ± 9.93-in the gas-hydrate region, 80.7 ± 5.4% of the initial concentration of hydrocarbons, respectively) (Figure 7).The difference between these values when calculating Student’s t coefficient did not show significant differences.

Given the significant difference between the frequency of detection of strains capable of only aerobic degradation of hydrocarbons in gas-hydrate and non-gas-hydrate area(in more than 20%), this feature is of geomicrobiological significance in identifying the type of gas deposit.

However, this part of the study revealed new properties of some of the tested strains.

Until the present study, there had been no published information on the ability of microorganisms of the genera *Stenotrophomonas*, *Psychrobacter*, *Micrococcus* and *Peribacillus* to oxidize oil hydrocarbons under aerobic and anaerobic conditions. For the other genera tested in this study, *Rhodococcus, Pseudomonas* and *Bacillus*, such an ability has already been described [13,23,39,40].

All representatives of the genus *Bacillus* tested by us had the ability to utilize oil, both under aerobic and anaerobic conditions. At the same time, in two strains, the degree of biodegradation of hydrocarbons under aerobic and anaerobic conditions did not have significant differences: in *Bacillus* sp. (in Bacteria) POI OP54-44/45-3(3), 87.82 ± 3.45 and 89.23 ± 2.72% of the initial concentration, respectively; *Bacillus* sp. (in Bacteria) POI LV81-19/135-3, 85.06 ± 2.59 and 83.58 ± 3.09% of the initial concentration, respectively. This genus also includes a single strain of *Bacillus* sp. (in Bacteria) POI OP54-28/198-6, in which the loss of hydrocarbons in the cultivation medium under aerobic conditions was higher than in anaerobic conditions (88.22 ± 2.72 and 80.95 ± 2.41%, of the initial concentration, respectively).

## 4. Discussion

An assessment of the relationship between the analyzed geomicrobiological indicators in the gas-hydrate region showed that the depth of the sampling station, as well as the detection of the bioindicator gene mcrA, made the lowest contribution to the characterization of the region.

**Figure 8:**
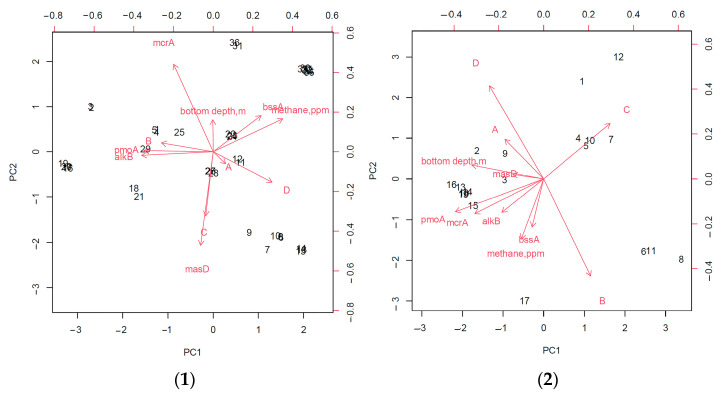
Relationships of the studied bioindicator signs in the gas-hydrate (**1**) and non-gas-hydrate (**2**) areas. (A) The degree of biodegradation of hydrocarbons under aerobic conditions; (B) the degree of biodegradation of hydrocarbons under anaerobic conditions; (C) frequency of isolation of strains; (D) frequency of isolation of strains capable of only aerobic degradation of hydrocarbons.

**Figure 9:**
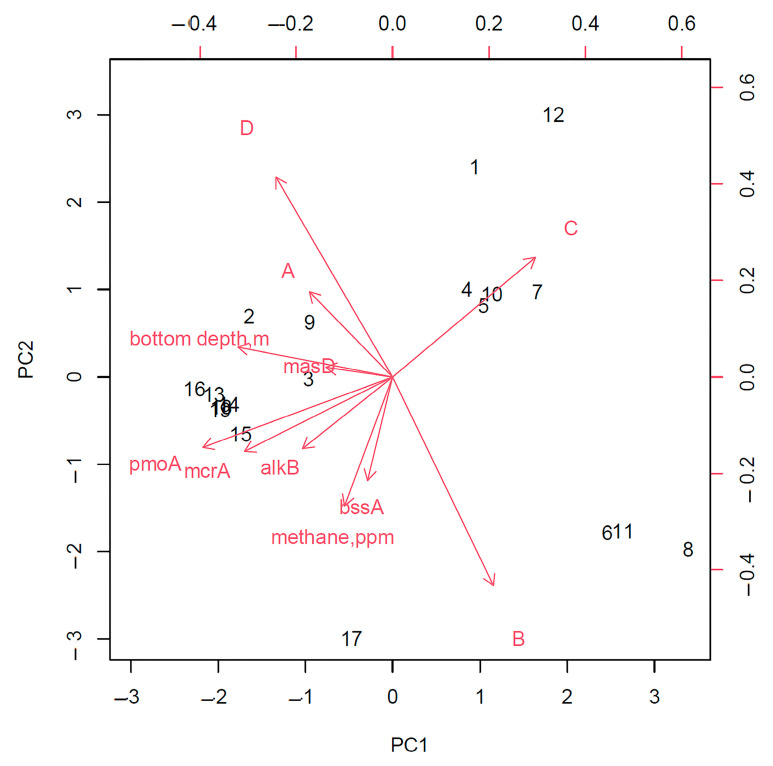
Relationships of the studied bioindicator signs throughout the study area without dividing into gas-hydrate and non-gas-hydrate zones. (A) The degree of biodegradation of hydrocarbons under aerobic conditions; (B) the degree of biodegradation of hydrocarbons under anaerobic conditions; (C) frequency of isolation of strains; (D) frequency of isolation of strains capable of only aerobic degradation of hydrocarbons.

An analysis of the principal components of the entire study area without dividing it into zones showed that the frequency of the detection of strains capable of only aerobic degradation of hydrocarbons and the presence of the gene for the anaerobic destruction of linear hydrocarbons, *masD*, are most closely related to the area where gas hydrates were found (Figure 8). In addition, this analysis made revealed a strong relationship between the detection of *pmoA* and *alkBB* genes, which can be explained by the fact that in areas of fluid discharge in gas fields, methane is often accompanied by heavier linear hydrocarbons [17,41]. In addition, the detection of the *bssA* gene was correlated with an increase in the intensity of the anaerobic oxidation of hydrocarbons (Figure 9).

The bacterial community of cultivated hydrocarbon-oxidizing bacteria in bottom sediments, according to the analysis of 16S rRNA, is formed by three phyla—*Pseudomonadota* (*Gammaproteobacteria*), *Bacillota* and *Actinomycetota*—which is confirmed by the literature data. The largest number of common species for the two study areas belongs to the phylum Pseudomonadota, class Gammaproteobacteria. It was shown that representatives of the genera *Pseudomonas*, *Psychrobacter* and *Stenotrophomonas* dominated in the studied samples of bottom sediments. It was established that representatives of the genus *Pseudomonas* (29.72%, or 11 out of 37 strains) predominated in the gas-hydrate region, and *Psychrobacter* (29.4%, or 5 out of 17 strains) dominated in the non-gas-hydrate region. Representatives of the genera *Bacillus*, *Pseudomonas*, *Stenotrophomonas* and *Psychrobacter* were found in all areas of the study. In addition, the bioindicative properties of some phyla were of greater importance. The confinement of representatives of the Nocardiaceae family of the phylum Actinomycetota to the areas where gas hydrates were found was revealed.

The analysis of the distribution of the studied strains to aerobic and anaerobic destruction and strains capable of only aerobic utilization did not reveal significantly different intensity degrees of biodegradation depending on the type of deposit.

As shown, ability to degradation of carbonaceous air under aerobic and/or anaerobic conditions depends on the genus of the studied strain, since all representatives of the genus *Bacillus* have the ability to utilize hydrocarbons both under aerobic and anaerobic conditions. In addition, there is a difference in the consumption of hydrocarbons in anaerobic conditions. Anaerobic degradation prevails to a greater extent in representatives of the genera *Pseudomonas* and *Rhodococcus* and to a lesser extent in *Stenotrophomonas* and *Bacillus*. The last genus belongs to the only strain in our study in which the ability for aerobic degradation dominated over anaerobic degradation by 7.27% (*Bacillus* sp. (in Bacteria) POI OP54-28/198-6).

## 5. Conclusions

In conclusion, the bioindicator signs we used made it possible to conduct geomi-crobiological mapping of the study area and show significant differences in the gas-hydrate and non-gas-hydrate areas. The differences primarily consisted of the detection of the anaerobic degradation gene and hydrocarbons, the presence of bioindicator species *Rhodococcus* sp., and the frequency of the detection of hydrocarbon-oxidizing bacteria capable of only the aerobic degradation of hydrocarbons.

Geomicrobiological indicators have previously been used only to describe the possible presence of an oil and gas reservoir. We propose to use them to describe the type of deposit, which significantly expands the possibility of using geomicrobiological exploration both for research and industrial purposes. Due to the fact that this comparison was carried out for the first time, the mechanisms of the processes that teach the differences between the gas-hydrate and non-gas-hydrate regions require further in-depth study.

The second important result was shown that the ability of hydrocarbon-oxidizing microorganisms to anaerobically utilize hydrocarbons has been greatly underestimated by previous studies. For the first time, we have revealed this ability for 38 out of 55 strains belonging to the genera *Stenotrophomonas*, *Psychrobacter*, *Micrococcus* and *Peribacillus*. The evaluation of the rate of utilization of oil hydrocarbons under anaerobic conditions by cultured methods made it possible to identify a large contribution of microorganisms to this process, which was not taken into account using standard biomarker genes. The anaerobic oxidation of hydrocarbon scan likely move not only through the well-known fumarate respiration pathway but also through other pathways: for example, with the help of a peroxide shunt. This mechanism allows for increasing the adaptive mechanisms of bacteria and enables aerobic microorganisms to exist under anaerobic conditions, which has been proven by the example of *Rhodococcus* both in our studies and in the literature [42]. An indirect confirmation of the widespread use of this mechanism is the predominance of the rate of hydrocarbon utilization under anaerobic conditions over aerobic conditions in the same strain, which is described in the literature [33].

Previous studies of aerobic and anaerobic oxidation of hydrocarbons have not been carried out in depth and only in individual model strains. Therefore, the relationship between the physiological and biochemical characteristics bacteria and the ability to utilize hydrocarbons under aerobic and anaerobic conditions is difficult to establish and requires further in-depth research. At the same time, the ecological role of this phenomenon is determined to be very large. That is why this work highlights the need for deeper study of the geomicrobiological processes of the aerobic and anaerobic oxidation of hydrocarbons in sea bottom sediments.

## Figures and Tables

**Table 1:** Characteristics of the samples selected for research in the gas-hydrate and non-gas-hydrate region in the northern part of the Sea of Japan.

Station	Latitude, N	Longitude, E	Bottom Depth, m	Sampling Depth, cm	Methane Concentration (Mean), ppm
Gas-hydrate area					
OP54-19	48°11.008′	141°02.322′	774	0–30	277.124
OP54-20a	48°18.699′	140°48.995′	269	0–30	72.782
OP54-22	48°15.600′	141°11.456′	718	0–30	221.563
OP54-28	47°43.279′	14°37.836′	907	0–30	101.223
OP54-29	47°48.892′	140°24.904′	658	0–30	105.359
OP54-33	47°33.125′	141°05.600′	1094	0–30	142.867
OP54-35	47°23.347′	141°32.411′	938	0–30	413.177
OP54-40	47°13.296′	139°37.080′	746	0–30	58.58
OP54-41	47°13.296′	139°37.080′	836	0–30	19.836
OP54-42	47°02.878	140°03.647′	1082	0–30	96.601
OP54-44	46°52.539′	140°29.752′	1094	0–30	91.29
OP54-59	46°54.715′	141°10.139′	864	0–30	0.002
Mean			831.67		122.13
Mean excluding max. value					97.87
Max. value			1094		413.18
Min. value			269		0.002
Non-gas-hydrate area					
LV81-01	43°25.359′	137°51.108′	3656	0–30	73.490
LV81-03	43°35.800′	137°25.400′	3670	0–30	11.484
LV81-07	43°57.442′	136°33.738′	1202	0–30	2.138
LV81-19	44°36.645′	136°32.382′	175	0–30	1.686
LV81-23	44°15.04′	137°24.493′	2681	0–30	0.0001
LV81-28	44°29.01′	137°37.0′	2260	0–30	1.256
LV81-30	44°39.957′	137°10.764′	1781	0–30	30.586
LV81-31	44°45.177′	136°58.049′	1328	0–30	5.125
LV81-34	46°13.850′	138°45.264′	1252	0–30	17.863
LV81-42	46°08.400′	138°43.100′	1381	0–30	26,389.516
LV81-51	45°54.800′	138°15.200′	851	0–30	28,978.270
LV81-57	45°33.700′	138°33.900′	1786	0–30	78.721
Mean			1835.25		4632.51
Mean excluding max. value					441.35
Max. value			3670		28,978.270
Min. value			175		0

**Table 2:** Oligonucleotide sequences of primers used in this study.

Target Compound	Gene	Sequence	Reference	Reaction Protocol
Indicator of aerobic destruction	*alkBB*	5′-GGTACGGSCAYTTCTACRTCGA-3′; 3′-CGGRTTCGCGTGRTGRT-5′	[28]	Initial denaturation, 5 min at 94 °C; 35 cycles of 30 s at 94 °C; 30 s at 60 °C; 30 s at 72 °C; final elongation, 8 min at 72 °C.
Indicator of anaerobic destruction	*masD*	5′-GGHMCVTDBGTVTGGAC-3′; 3′-RTCRTCRTTDCCCCAYTTNGG-5′	[21]	Denaturation, 15 min at 95 °C; 55 cycles of 30 s at 95 °C; 30 s at 54 °C; 30 s at 72 °C; final elongation, 10 min at 72 °C.
Indicator of anaerobic destruction of aromatic hydrocarbons	*bssA*	5′-ACGACGGYGGCATTTCTC-3′, 3′-GCATGATSGGYACCGACA-5′	[30]	Denaturation, 5 min at 94 °C; 35 cycles of 45 s at 94 °C; 30 s at 55 °C; 60 s at 72 °C; final elongation, 10 min at 72 °C.
Methanotrophic bacteria gene	*pmoA*	189F-5′-GGNGACTGGGACTTCTGG-3′, 682R-5′-GAASGCNGAGAAGAASGC-3′	[22]	Initial denaturation, 3 min at 94 °C; 35 cycles of 30 s at 94 °C; 30 s at 47.7 °C; 45 s at 72 °C; final elongation, 5 min at 72 °C.
Methanogenic microorganisms gene	*mcrA*	5′-TAYGAYCARATHTGGYT-3′ 5′-ACRTTCATNGCRTARTT-3′	[31]	Initial denaturation, 3 min at 94 °C; 35 cycles of 40 s at 94 °C; 45 s at 53 °C; 90 s at 72 °C; final elongation, 10 min at 72 °C.

**Table 3:** Values of the main diversity indices in the studied areas of the northern part of the Sea of Japan.

Region/Index	Gas-Hydrate Region	Non-Gas-Hydrate Region
Shannon species diversity index	1.84	1.88
Simpson dominance index	0.19	0.18
Jaccard coefficient	0.17	

## Data Availability

Not applicable.

## Supplementary Materials

The following supporting information can be downloaded at: https://www.mdpi.com/article/10.3390/biology11121802/s1, Figure S1: Phylogenetic tree built on the basis of sequence analysis of 16S rRNA gene fragments of bacteria isolated from bottom sediments of the northern part of the Sea of Japan (gas-hydrate area). Scale bar length: 1 substitution per 100 nucleotides. Evolutionary distances were computed according to maximum-likelihood phylogenetic tree based on 16S rRNA gene. The reliability of each branch was evaluated by bootstrap analysis based on 1000 replications. Figure S2: Phylogenetic tree built on the basis of sequence analysis of 16S rRNA gene fragments of bacteria isolated from bottom sediments of the northern part of the Sea of Japan (non-gas-hydrate area). Scale bar length: 1 substitution per 100 nucleotides. Evolutionary distances were computed according to maximum-likelihood phylogenetic tree based on the 16S rRNA gene. The reliability of each branch was evaluated by bootstrap analysis based on 1000 replications. Table S1: Composition of media used. Table S2: Degree of biodegradation of hydrocarbons by strains isolated from bottom sediments of gas-hydrate area under aerobic and anaerobic conditions. Table S3: Degree of biodegradation of hydrocarbons by strains isolated from bottom sediments of non-gas-hydrate area under aerobic and anaerobic conditions.
