# Supplementary material for: Groups of Geomicrobiological Indicators Are Spread across Gas-Hydrate and Non-Gas-Hydrate Areas in the Northern Part of the Sea of Japan
Source: Biology (Basel). 2022 Dec 12;11(12):1802. doi: 10.3390/biology11121802 (PMC9774978; doi:10.3390/biology11121802)
Supplement: Supplementary file 1 [file biology-11-01802-s001.zip › biology-1978051-supplementary.pdf]

Table S1. Additional materials 1. Composition of media used.

Microbiological Media	Media composition (g/l)
marine ammonium medium 1313 (Marine ammonium mineral salts)	NaCl - 20, $(NH_4)_2SO_4$ -1, $CaCl_2$ • $2H_2O$ - 0.2, $MgSO_4$ • $7H_2O$ - 1 , $FeSO_4$ • $7H_2O$ - 0.002, Na_2WO_4 • $2H_2O$ - 0.003, Na_2MoO_4 • $2H_2O$ - 0.020, microelement solution SL-10 - 1, KH_2PO_4 - 0.36, K_2HPO_4 - 2.34, pH 7.2
marine mineral medium (Marine salt medium)	KH_2PO_4 - 0.5, NaH_2PO_4 • H_2O - 0.5, $MgSO_4$ • $7H_2O$ - 0.5, NaCl - 4.0, NH_4Cl - 0.5, microelement solution SL-10 – 1, pH 7.2
a modified Voroshilova-Dianova medium	K_2HPO_4– 1.0; KH_2PO_4 – 1.0; NH_4NO_3 – 1.0; $MgSO_4$ • $7H_2O$ – 0.2; $CaCl_2$ • $2H_2O$ – 0.02, a solution of trace elements SL-10, a solution of vitamins according to Volin, $FeCl_2$-2 drops of a concentrated solution. Artificial seawater (g/l) was used as the basis for the medium: NaCl – 27.5, $MgCl_2$ – 5.0, $MgSO_4$•$7H_2O$ – 2.0, $CaCl_2$– 0.5, KCl – 1.0, $FeSO_4$ – 0.001[30], pH 7.0-7.2.

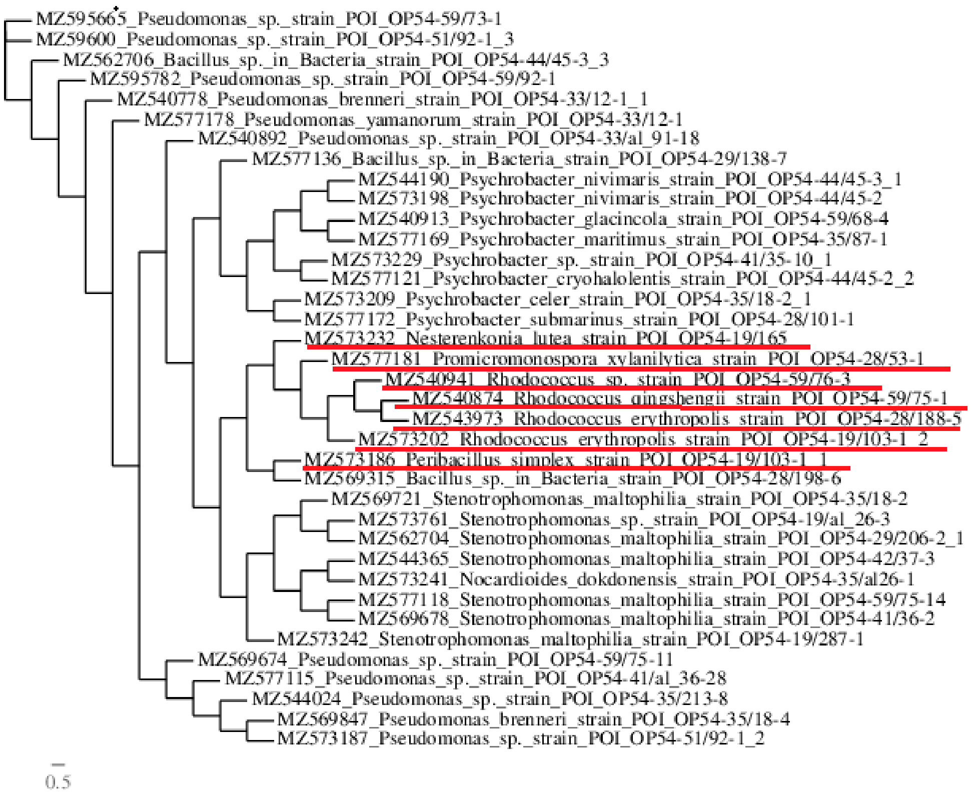

Figure S1. Phylogenetic tree built on the basis of sequence analysis of 16S rRNA gene fragments of bacteria isolated from bottom sediments of the northern part of the Sea of Japan (gas-hydrate area). Scale bar length: 1 substitution per 100 nucleotides. Evolutionary distances were computed according to Maximum-likelihood phylogenetic tree based on 16S rRNA gene. The reliability of each branch was valuated by bootstrap analysis based on 1000 replications.

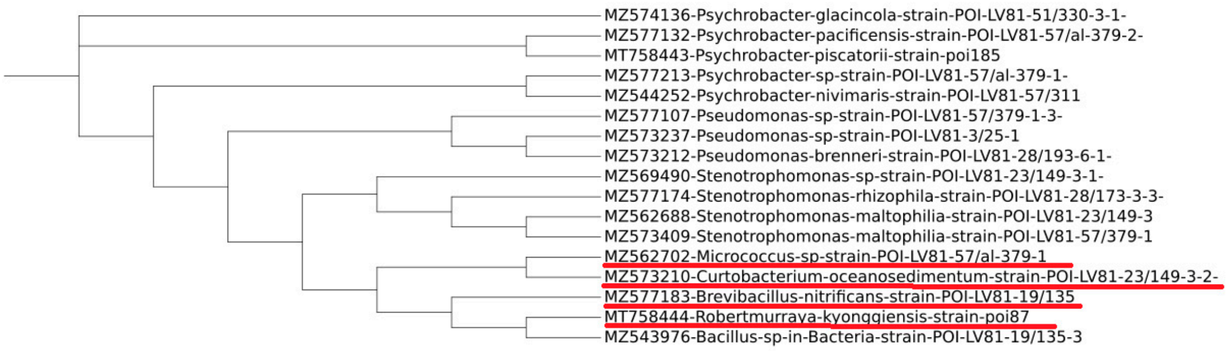

Figure S2. Phylogenetic tree built on the basis of sequence analysis of 16S rRNA gene fragments of bacteria isolated from bottom sediments of the northern part of the Sea of Japan (Non-gas-hydrate area). Scale bar length: 1 substitution per 100 nucleotides. Evolutionary distances were computed according to Maximum-likelihood phylogenetic tree based on 16S rRNA gene. The reliability of each branch was valuated by bootstrap analysis based on 1000 replications.

Table S2. Degree of biodegradation of hydrocarbons by strains isolated from bottom sediments of gas-hydrate area under aerobic and anaerobic conditions.

Strain code and NCBI number	The degree of biodegradation of hydrocarbons	
	under aerobic conditions	under anaerobic conditions
MZ573202 Rhodococcus erythropolis strain POI OP54-19/103-1(2)	78,71±1,94	
MZ540874 Rhodococcus qingshengii strain POI OP54-59/75-1	84,50±3,29	97,13±2,90
MZ540941 Rhodococcus sp. strain POI OP54-59/76-3	63,27±2,22	94,46±2,81
MZ543973 Rhodococcus erythropolis strain POI OP54-28/188-5	89,56±3,25	97,44±3,83
MZ577118 Stenotrophomonas maltophilia strain POI OP54-59/75-14	72,31±2,24	
MZ544365 Stenotrophomonas maltophilia strain POI OP54-42/37-3	63,51±1,95	
MZ573242 Stenotrophomonas maltophilia strain POI OP54-19/287-1	81,79±2,65	80,13±2,84
MZ573761 Stenotrophomonas sp. strain POI OP54-19/al 26-3	69,54±2,51	79,54±2,80
MZ562704 Stenotrophomonas maltophilia strain POI OP54-29/206-2(1)	51,98±1,77	84,74±2,43
MZ569721 Stenotrophomonas maltophilia strain POI OP54-35/18-2	97,38±3,83	100,00±3,77
MZ569678 Stenotrophomonas maltophilia strain POI OP54-41/36-1	84,02±2,61	94,64±3,33
MZ577121 Psychrobacter cryohalolentis strain POI OP54-44/45-2 (2)	75,71±2,78	
MZ573209 Psychrobacter celer strain POI OP54-35/18-2(1)	73,32±2,70	
MZ577169 Psychrobacter maritimus strain POI OP54-35/87-1	96,24±3,69	
MZ573198 Psychrobacter nivimaris strain POI OP54-44/45-2	78,18±2,69	92,30±3,60
MZ573229 Psychrobacter sp. strain POI OP54-41/35-10(1)	72,53±2,40	91,09±3,28
MZ540913 Psychrobacter glacincola strain POI OP54-59/68-4	79,98±2,85	98,73±3,10
MZ544190 Psychrobacter nivimaris strain POI OP54-44/45-3(1)	87,54±2,57	90,40±3,09
MZ577172 Psychrobacter submarinus strain POI OP54-28/101-1	86,89±3,40	97,28±3,33
MZ569847 Pseudomonas brenneri strain POI OP54-35/18-4	77,81±2,76	
MZ540778 Pseudomonas brenneri strain POI OP54-33/12-1(1)	76,84±2,54	93,17±2,92
MZ540892 Pseudomonas sp. strain POI OP54-33/al 91-18	83,83±3,20	96,77±3,25
MZ544024 Pseudomonas sp. strain POI OP54-35/213-8	56,07±1,71	89,33±2,58
MZ595665 Pseudomonas sp. strain POI OP54-59/73-1	77,12±2,89	84,89±2,51
MZ595782 Pseudomonas sp. strain POI OP54-59/92-1	70,53±2,16	97,22±3,62
MZ59600 Pseudomonas sp. strain POI OP54-51/92-1(3)	81,15±3,19	86,61±3,25
MZ577115 Pseudomonas sp. strain POI OP54-41/al 36-28	76,39±2,58	95,29±3,46
MZ569674 Pseudomonas sp. strain POI OP54-59/75-10	73,25±3,13	94,77±2,97
MZ573187 Pseudomonas sp. strain POI OP54-51/92-1(2)	67,37±2,40	85,01±2,96
MZ577178 Pseudomonas yamanorum strain POI OP54-33/12-1	68,01±1,09	84,91±2,13
MZ595666 Bacillus sp. (in: Bacteria) strain POI LV81-57/379-1(1)	71,18±2,30	92,00±2,90
MZ562706 Bacillus sp. (in: Bacteria) strain POI OP54-44/45-3(3)	87,82±3,45	89,23±2,72
MZ577136 Bacillus sp. (in: Bacteria) strain POI OP54-29/138-6	68,21±2,46	86,01±2,60
MZ569315 Bacillus sp. (in: Bacteria) strain POI OP54-28/198-6	88,22±2,72	80,95±2,41
MZ573241 Nocardioides dokdonensis strain POI OP54-35/al26-1	79,98±2,52	
MZ573232 Nesterenkonia lutea strain POI OP54-19/165	73,69±2,29	
MZ573186 Peribacillus simplex strain POI OP54-19/103-1(1)	87,64±3,45	92,40±2,66

Table S3. Degree of biodegradation of hydrocarbons by strains isolated from bottom sediments of non- gas-hydrate area under aerobic and anaerobic conditions.

Strain code and NCBI number	The degree of biodegradation of hydrocarbons	
	under aerobic conditions	under anaerobic conditions
MZ577132 Psychrobacter pacificensis strain POI LV81-57/al 379 (2)	80,21±2,75	
MZ577174 Stenotrophomonas rhizophila strain POI LV81-28/173-3(3)	86,33±2,67	
MZ573210 Curtobacterium oceanosedimentum strain POI LV81-23/149-3(2)	75,56±2,31	
MZ573212 Pseudomonas brenneri strain POI LV81-28/193-6(1)	70,33±2,72	92,35±3,27
MZ573237 Pseudomonas sp. strain POI LV81-3/25-1	53,07±2,08	76,62±2,22
MZ543976 Bacillus sp. (in: Bacteria) strain POI LV81-19/135-3	85,06±2,59	83,58±3,09
MZ544252 Psychrobacter nivimaris strain POI LV81-57/311	69,49±2,13	77,22±2,34
MZ573409 Stenotrophomonas maltophilia strain POI LV81-57/379-1	81,81±2,58	100,00±3,83
MZ562702 Micrococcus sp. strain POI LV81-57/al 379-1	78,77±2,76	72,34±2,56
MZ574136 Psychrobacter glacincola strain POI LV81-51/330-3(1)	77,34±2,89	88,26±3,35
MZ577107 Pseudomonas sp. strain POI LV81-57/379-1(3)	53,25±1,68	69,53±2,07
MZ569490 Stenotrophomonas sp. strain POI LV81-23/149-3(1)	72,56±2,59	88,03±2,57
MT758443 Psychrobacter piscatorii strain poi185	75,3±2,11	91,1±1,09
MZ577213 Psychrobacter sp. strain POI LV81-57/al 379 (1)	72,3±1,98	84,1±3,27
MZ562688 Stenotrophomonas maltophilia strain POI LV81-23/149-3	63,2±1,37	86,9±2,07
MZ577183 Brevibacillus nitrificans strain POI LV81-19/135	54,6±3,16	87,3±1,91
MT758444 Robertmurraya kyonggiensis strain poi87	75,1±2,09	94,2±2,01
